# Which Type of the Promising COVID-19 Vaccines Produces Minimal Adverse Effects? A Retrospective Cross-Sectional Study

**DOI:** 10.3390/vaccines10020186

**Published:** 2022-01-25

**Authors:** Heba M. Attash, Luma M. Al-Obaidy, Harith Kh. Al-Qazaz

**Affiliations:** Department of Clinical Pharmacy, College of Pharmacy, University of Mosul, Ninevah 81011, Iraq; heba.attash@uomosul.edu.iq (H.M.A.); al_qazaz73@uomosul.edu.iq (H.K.A.-Q.)

**Keywords:** COVID-19, AstraZeneca, Pfizer, Sinopharm, post-vaccine symptoms, health care workers

## Abstract

Since the declaration of Coronavirus-2019 (COVID-19) as a pandemic by the World Health Organization (WHO), it was clear that vaccination is the best way to overcome it. Sinopharm, AstraZeneca and Pfizer were the first vaccines introduced to defeat it. To recognize the short-term adverse effects among Iraqi health care workers (HCWs) after vaccination, the three COVID-19 vaccines that are currently available in Iraq were compared. An online survey was distributed to Iraqi HCWs who had received at least one of the COVID-19 vaccines as part of a retrospective cross-sectional study. Data were statistically analyzed using SPSS. The total number of participants was 843. The majority of the participants (85.9%) were under 39 years old, with 78.8% of them being females. Around 60% of individuals had received the Pfizer vaccine. Severe acute respiratory syndrome coronavirus 2 (SARS-CoV-2) had infected 46.7% of the total participants. A total of 628 out of 843 participants experienced adverse effects after receiving the vaccine, accounting for 74.49% of the overall respondents. After receiving the COVID-19 vaccine, the vast majority of respondents who received the three vaccines experienced pain at the injection site (*n* = 800), while other side effects like headache, myalgia, tiredness and fever mainly appeared with Pfizer and AstraZeneca vaccines. Most of the reported adverse effects were tolerable and self-limited and they were linked to the AstraZeneca and Pfizer vaccines.

## 1. Introduction

The first case of SARS-CoV-2 was reported in China at the end of 2019 and rapidly spread to other countries causing a global pandemic all over the world [1]. Although many medications, herbal and chemical compounds have been used to fight COVID-19, they have only a supportive role in the management of SARS-CoV-2 cases. As a consequence of the pandemic, scientists and academic researchers from the United Kingdom, China, the United States and other countries were attempting to introduce some promising vaccines for COVID-19 [2]. The successful development of vaccines is probably the greatest goal for the prevention of this pandemic.

In December 2020, the World Health Organization (WHO) announced the release of the first COVID-19 vaccines [3]. Because HCWs were particularly exposed to the biological dangers of COVID-19, several governmental institutions identified them the first group that should get the vaccine [4]. COVID-19 vaccines have been developed at an extraordinary speed. As an extension of this rapid speed of vaccine production, vaccine safety issues and post-marketing safety monitoring problems were generated [5]. Each of these vaccines has different targets on the coronavirus. SARS-CoV-2 is an enveloped virus including a single-stranded, positive-sense ribonucleic acid (RNA) genome (~30,000 nucleotides) containing spike proteins on it’s surface, similar to other Corona viruses [6]. The Pfizer-BioNTech vaccine (COMIRNATY) is a messenger RNA (mRNA) vaccine encoding the viral spike S glycoprotein of SARS-CoV-2. It induces a protective immune response and reduces the incidence of future COVID-19 [7,8]. Vaxzevria (previously COVID-19 Vaccine AstraZeneca) is made up of a chimpanzee adenovirus virus that has been modified to contain the spike S1 gene (*ChAdOx1-S*). The vaccine is produced in genetically modified human embryonic kidney (HEK) 293 cells and by recombinant DNA technology [9]. China produced a vaccine called Sinopharm, which was an inactivated vaccine consisting of viruses that were treated by physicochemical methods to reduce their pathogenicity [10]. The immune system will be prepared for the future attack by forming antibodies against the inactivated viruses [11]. The development of immunity following a vaccine will generate side effects [8]. In enhancing safety, it is critical to identify and report the side effects of vaccinations. Fever, redness, swelling at the injection site and anaphylaxis are all possible side effects associated with vaccine administration [2]. One of the countries most affected by the pandemic was Iraq. The number of infected cases was more than 1 million and more than fifteen thousand deaths were associated with COVID-19 [12]. Iraqi people started to get the COVID-19 vaccine in March 2021. After electronic registration, people started to get the COVID-19 vaccine in hospitals. The three vaccines available were the Sinopharm, Oxford-AstraZeneca, and Pfizer-BioNTech ones.

A study conducted by Hatmal et al., 2021 in Jordan, to report the incidence of adverse effects among general populations after receiving different vaccines, concluded that the majority of adverse effects were minor to moderate and they were well tolerated and expected as a result of building immunity by the body [5]. In another study conducted by El-Shitany et al., 2021 in Saudi Arabia, several side effects were reported, some of them at the injection site while the others at the whole body like site pain, fever, joint pain, difficulty in breathing, headache, tiredness, chills, drowsiness and palpitation especially among elderly and rare side effects like Bell’s palsy and lymphadenopathy were also recognized [8]. In a study conducted in the Czech Republic to report side effects of the Pfizer vaccine among HCWs, they found that majority of participants had minor side effects like pain at the site of injection, chills, headache, fatigue and muscle pain and these side effects were more common among individuals who had received two doses of the vaccine [13].

There are limited studies on experience with side effects reactions after receiving first or second doses of COVID-19 vaccines, therefore, this study aimed to discover the type, prevalence and intensity of the adverse reactions experienced by Iraqi HCWs after receiving a first or second dose of COVID-19 vaccines, and to compare side effects between three different vaccines.

## 2. Materials and Methods

A retrospective cross-sectional web-based survey was used. The convenience sampling method was used to recruit the participants of this study, who were Iraqi HCWs (physicians, pharmacists, dentists) from 27 September to 7 October 2021. The inclusion criteria were vaccinated Iraqi HCWs. Exclusion criteria were non vaccinated HCWs as well as other general populations. A self-administered survey designed in Google Forms was accessible online during the data collection period. Each member setting was modified such that they will only submit one reply; responses were double-checked to make sure that no answers were duplicated. The survey was sent to a variety of groups by websites and online methods (social media like Messenger and Facebook). The respondents were asked to state any side effects experienced from the COVID-19 vaccination. Part one of the online survey contained demographic information of the respondents (sex, age, employment, type and dose of vaccine, prior infection with COVID-19) as well as the development of any side effects following vaccination. Part two consisted of closed questions (multiple-choice items) about the common local and systemic adverse effects of the vaccine in addition to these closed questions, one open question was used to record other side effects, and other questions were added to report if there is any analgesic or medication used to treat these side effects. The study’s protocol was authorized by a Collegiate Committee for Medical Research Ethics at the University of Mosul’s CCMRE-phA-21-5. Before answering the questionnaire, the respondents were informed that their participation is voluntary and that their consent was required. Google form was used to collect data, while Excel 10 (Microsoft, Albuquerque, NM, USA) and Statistical Package for Social Sciences version 25 (IBM SPSS Statistics for Windows, IBM Corp., Armonk, NY, USA) were used for entering and coding data. Different demographic characteristics (age, gender, profession, years of experience, type of vaccine, number of doses, onset of symptoms) were compared using descriptive statistics (means and standard deviation). Chi-square test and Fisher exact test were used to calculate the distribution of adverse effects, type of analgesic used, and physician consultation among the three vaccine group recipients. A *p*-value of less than 0.05% is considered statistically significant.

## 3. Results

### 3.1. Basic Demographic Characteristics of Study Participants

A total sample of 843 respondents was reached with the mean age (SD) of the study population was 32.39 (6.88) and the mean years of experience was 7.96 (6.79). Among the total respondents, there were 664 (78.8%) females and 179 (21.2%) males. Doctors represented the highest respondents to the study survey (415, 49.2%) and 665 (78.9%) of the total sample had a bachelor degree. Most of the respondents had received their second dose of the vaccine 651 (77.2%) and the Pfizer type of vaccine was the most used one among the study population (502, 59.5%) with around the same proportion for both AstraZeneca 187 (22.2%) and Sinopharm 154 (18.3%). Less than half of the participants were previously infected with SARS-CoV-2 before being vaccinated. Table 1 shows the basic demographic characteristics of the study sample.

### 3.2. Adverse Effects to Vaccines

All respondents were asked if they suffered from any adverse effects after they got their vaccine. Out of the 843 respondents, only 215 (25.5%) indicated that they did not suffer from any adverse effects. However, a majority of them reported pain at the injection site. Table 2 show the frequency of each reported adverse effect among the three types of vaccines documented to be used in the country. A significant association between vaccine type and occurrence of adverse effects was found, where AstraZeneca and Pfizer were found produce more adverse effects (84.5% and 75.3%, respectively). From Table 2 and the Chi-square test, Pfizer followed by AstraZeneca vaccines were reported to have a higher frequency of adverse effect or severity rather than Sinopharm vaccine. A higher frequency of moderate to severe pain at the site of injection was reported among those who received AstraZeneca and Pfizer vaccine (*p* ˂ 0.05). Concerning dermal reactions, including redness, swelling and skin rash, these were also presented more among subjects who received the AstraZeneca and Pfizer vaccines (*p* ˂ 0.05), nevertheless, the association of skin rash with vaccine type was not significant.

In terms of systemic reactions including tiredness, headache, myalgia, fever and chills; higher frequencies were significantly found among subjects who received the AstraZeneca and Pfizer vaccines (*p* ˂ 0.05). With regards to gastro-intestinal (GIT) symptoms, (nausea, vomiting, diarrhea), the AstraZeneca vaccine was associated with a higher rate of all symptoms, however, diarrhea was found to not be significantly associated with vaccine type. Mild to moderate palpitation were significantly associated with vaccine type and higher frequency found in Pfizer followed by AstraZeneca receiver (*p* ˂ 0.05). Reported difficulty in breathing and sore throat the lowest among Sinopharm vaccines (*p* ˂ 0.05).

### 3.3. Medical Seeking

All respondents were asked about the type of analgesic used to treat their symptoms and if they consulted physicians about their adverse reaction after vaccination. Table 3 shows the frequencies of all analgesics reported to have been used where paracetamol was the most used analgesics. A small proportion of respondents were reported that they consulted physicians for their symptoms, however, the study sample was a medical staff population and this may explain the low rate of physician consultations.

## 4. Discussion

This study aimed to determine the type, prevalence and intensity of adverse effects associated with COVID-19 vaccines among HCWs after receiving a first or second dose of the vaccines and to make a comparison between the three available vaccines. Vaccinated HCWs from several cities in Iraq participated in this study. The current study’s findings are based on an online questionnaire that asked about COVID-19 vaccines adverse effects from September to October 2021. According to the data, the most of respondents were females as due to their better scores on high school bachelor tests, females constituted a large proportion of students in Iraq’s medical colleges from 2003 [14]. The majority of our participants had received the Pfizer vaccine, while the Pfizer-BioNTech vaccine was the third most commonly received by participants in Jordan [5].

According to phase III clinical trials including 43,538 individuals, the vaccination was reported to be 95% effective in reducing during the first 28 days. Both European Medicines Agency (EMA) and Food and Drugs Administration (FDA) licensed the use of the Pfizer vaccine and is now available in many states including the European Union, the United Kingdom and the United States [10]. Moreover, although Pfizer has not disclosed any major safety issues to date, there are still doubts about its medium or long-term safety [15].

Ghazi et al., studied the acceptance of the COVID-19 vaccine in Iraq, about half of the participants preferred AstraZeneca, while small proportions of the Iraqi populations preferred the Chinese vaccine, however, the Pfizer vaccine was not included in the study [16]. There was a significant association between vaccine type and prevalence of adverse effects with AstraZeneca and Pfizer having the highest rates of adverse effects in comparison to the Sinopharm vaccine. While the production and storage of DNA and RNA vaccines are simple in addition to the good stability and low toxicity, issues affecting their immunogenicity and safety need to be investigated extensively [17]. Most of the adverse effects were observed after the first dose, which was similar to the findings of Hatmal et al., in Jordan, whereas El-Shitany et al., noticed that most of the adverse effects were occurred after the second dose, Polack et al., observed that the prevalence of symptoms was similar after first and second doses [18]. According to the Centers for Disease Control and Prevention (CDC), the severity of symptoms increased after the second dose [5].

Those who received AstraZeneca and Pfizer vaccines showed an increased incidence of moderate to severe pain at the injection site (*p* ˂ 0.05). The results of the current study matched those of the Jordanian study which found that injection site pain was more prevalent in people who received the Pfizer vaccine, then the AstraZeneca one, and finally the Sinopharm product. According to a study conducted in Iraq [12], pain at the injection site was similarly more common among Pfizer participants. Redness, swelling, skin rash and other dermal reactions were more common among subjects who received the AstraZeneca and Pfizer vaccines. However, there was no significant association between vaccine type and skin rashes. The immune system response can explain this finding, as the immune system may release cytokines which can cause inflammation in muscles, blood vessels, and other tissues which may cause flu-like symptoms that continue for several days following vaccination [8]. Dermatological symptoms were infrequent and non-significant according to studies done in Saudi Arabia and Jordan [5,8].

Concerning systemic effects like headache and tiredness, individuals who received the Pfizer and AstraZeneca vaccines reported significantly greater frequencies (*p* < 0.05). Over half of those who received the AstraZeneca and Pfizer vaccines experienced headaches, and the results were similar for persons who received the Pfizer vaccine in Saudi Arabia [8]. The same results were also reported by Polak et al., among participants who received the Pfizer vaccine in more than 150 areas around the world [18]. Headache was a prevalent adverse effect among the individuals in the Jordan study who received the three types of vaccination [5].

In terms of GIT symptoms, (nausea, vomiting and diarrhea) the AstraZeneca vaccine was linked to a greater incidence of all symptoms apart from diarrhea, which was not shown to be significantly associated with vaccine type. Findings similar to ours were reported in the Jordanian study, which showed a greater rate of all GIT symptoms apart from diarrhea [5]. GIT symptoms were rare among Pfizer vaccination recipients in a Saudi Arabia study [8].

A higher frequency of mild to moderate palpitation was shown to be significantly associated with vaccination type, with Pfizer showing the highest frequency (*p* ˂ 0.05), followed by AstraZeneca. Palpitation was uncommon among persons who receive the Pfizer vaccine in Saudi Arabia [8]. Difficulty in breathing was very low among Sinopharm recipients (*p* < 0.05). Difficulty in breathing was more frequent among AstraZeneca vaccine recipients in the additional study conducted in Iraq [12], while in Saudi Arabia the percentage of individuals who suffered from it was rare [8]. In a study done in Indonesia, to examine the incidence of adverse effects after vaccination with COVID-19 vaccine among hospital staff, breathlessness was also very rare (about 1%) [19]. Regarding sore throat, it was very low among Sinopharm recipients. Moreover, sore throat was also shown to be rare among Pfizer vaccine recipients in the Jordan study [5].

Other rare side effects include insomnia, lymphadenopathy, somnolence, metallic taste, joint pain, back pain. Lymphadenopathy also was identified among persons who receive the Pfizer vaccine in a Saudi Arabian study [8]. In a study conducted in Jordan, half of those who received Pfizer and Sinopharm vaccines complained of joint pain, as did most of those who received the AstraZeneca vaccine [5], and body and joint pain were observed by 12.52% of individuals In Bangladesh, patients received only the AstraZeneca vaccine [20]. In Saudi Arabia, around 2% of people who received the Pfizer and AstraZeneca vaccines suffered from joint or bone pain [1].

In the Czech study, where patients received only the Pfizer vaccine, more than a quarter of the participants suffered from joint pain, while 16% reported lymphadenopathy [13]. Lymphadenopathy was considered one of the unintended adverse effects according to a FDA report involving 64 cases; nevertheless, it would have been being an expected adverse effect, because it is prevalent in some other types of vaccines like the influenza vaccine and the human papilloma virus vaccine [21].

In the Czech study, there were reports of taste disturbances [13]. In another study conducted in Iraq, in which the three types of vaccines were also used, some individuals complained of a loss of smell and taste [12]. In India, sleeplessness was noted in eight individuals who received Sinopharm and Pfizer vaccinations [22]. Considering the use of analgesics to relieve symptoms, the majority of participants used paracetamol. In India, more than half of people used paracetamol to relieve symptoms [22]. Despite that the use of paracetamol to relieve post-vaccination symptoms is safe, pain medications are not suggested as a normal preventive measure because there is evidence of weakened immune response as a possible consequence [23].

## 5. Limitation of the Study

Although this study involved a large number of participants in different Iraqi cities and it was one of few studies that deal with the adverse effects of COVID-19 vaccines in Iraq, the participants involved were HCWs, therefore the adverse effects were probably accurately reported. The study has some limitations. First, this study is a descriptive cross-sectional study using an online survey, so only individuals who frequently used the internet participated in the study. The participation of the old age group was low. This study also used subjective measures and was based on trust. A face to face interview is preferred to collect an accurate results. We only studied the short-term adverse effects of the vaccines, and a prospective long duration follow up study in the general population is recommended. A specific test like D-dimer could be involved to measure the rare side effects associated with the AstraZeneca vaccine, and also a more comprehensive study involving all authorized vaccines is recommended.

## 6. Conclusions

This study was conducted to evaluate the incidence of adverse effects among HCWs after receiving Pfizer, AstraZeneca, and Sinopharm vaccines in Iraq. We noted that the majority of individuals who received one of the three vaccines reported pain at the injection site. Headache, myalgia, tiredness and fever were reported among individuals who received the Pfizer and AstraZeneca vaccines. In general, just a few individuals needed to see a physician, because the adverse effects were minimum and well tolerated. Paracetamol was the most commonly used analgesic to treat adverse effects. Among the three types of vaccines, the Sinopharm one produced the lowest number of adverse effects.

## Figures and Tables

**Table 1:** Basic demographic characteristics of study participants.

Variable	*n*	%
Age		
20–29 years	341	40.5
30–39 years	383	45.4
40 years and older	119	14.1
Gender		
Male	179	21.2
Female	664	78.8
Job		
Doctors	415	49.2
Dentist	192	22.8
Pharmacists	236	28.0
Graduation level		
Graduated	665	78.9
Postgraduates	178	21.1
Years of Experience		
Less than 5 years	315	37.4
5–9 years	229	27.2
10–14 years	170	20.2
15–19 years	57	6.8
20 years and more	72	8.5
Vaccination status		
First dose only	192	22.8
Two doses	651	77.2
Type of vaccine		
Sinopharm	154	18.3
AstraZeneca	187	22.2
Pfizer	502	59.5
Symptoms		
Yes	628	70.6
No	215	29.4
Onset of symptoms		
No symptoms	215	25.5
After 1st vaccine dose	271	32.1
After 2nd dose	123	14.6
After 1st and 2nd dose	234	27.8
History of previous infection		
Infected	394	46.7
Not infected	449	53.3
Corticosteroid use (*n* = 635)		
Yes	66	7.8
No	569	67.5

**Table 2:** Adverse effect distribution among the study group.

Variable	Vaccine Type			*p*-Value
	Sinopharm	AstraZeneca	Pfizer	
	154 (18.3%)	187 (22.2%)	502 (59.5%)	
Adverse effect after vaccination				0.000
Yes	59 (38.3)	158 (84.5)	378 (75.3)	
No	95 (61.7)	29 (15.5)	124 (24.7)	
Pain				0.000
None	28 (18.2)	4 (2.1)	11 (2.2)	
Mild	81 (52.6)	47 (25.1)	91 (18.1)	
Moderate	41 (26.6)	101 (54.0)	246 (49.0)	
Sever	4 (2.6)	35 (18.7)	154 (30.7)	
Dermatological				
Redness				0.000
None	129 (83.8)	118 (63.1)	377 (75.1)	
Mild	24 (15.6)	52 (27.8)	96 (19.1)	
Moderate	1 (0.6)	15 (8.0)	20 (4.0)	
Sever	0 (0.0)	2 (1.1)	9 (1.8)	
Swelling				0.000
None	127 (82.5)	96 (51.3)	290 (57.8)	
Mild	21 (13.6)	64 (34.2)	145 (28.9)	
Moderate	6 (3.9)	23 (12.3)	56 (11.2)	
Sever	0 (0.0)	4 (2.1)	11 (2.2)	
Skin rash				0.507 **
None	150 (97.4)	183 (97.9)	482 (96.0)	
Mild	3 (1.9)	3 (1.6)	14 (2.8)	
Moderate	1 (0.6)	1 (0.5)	1 (0.2)	
Sever	0 (0.0)	0 (0.0)	5 (1.0)	
Systemic reactions				
Tiredness				0.000
None	73 (47.4)	27 (14.4)	110 (21.9)	
Mild	54 (35.1)	32 (17.1)	122 (24.3)	
Moderate	22 (14.3)	72 (38.5)	163 (32.5)	
Sever	5 (3.2)	56 (29.9)	107 (21.3)	
Headache				0.000
None	88 (57.1)	50 (26.7)	198 (39.4)	
Mild	43 (27.9)	42 (22.5)	125 (24.9)	
Moderate	17 (11.0)	53 (28.3)	120 (23.9)	
Sever	6 (3.9)	42 (22.5)	59 (11.8)	
Myalgia				0.000
None	88 (57.1)	31 (16.6)	148 (29.5)	
Mild	44 (28.6)	45 (24.1)	129 (25.7)	
Moderate	18 (11.7)	67 (35.8)	135 (26.9)	
Sever	4 (2.6)	44 (23.5)	90 (17.9)	
Chills				0.000
None	133 (86.4)	74 (39.6)	299 (59.6)	
Mild	11 (7.1)	50 (26.7)	98 (19.5)	
Moderate	7 (4.5)	36 (19.3)	60 (12.0)	
Sever	3 (1.9)	27 (14.4)	45 (9.0)	
Fever				0.000
None	115 (74.7)	41 (21.9)	183 (36.5)	
Mild	24 (15.6)	37 (19.8)	137 (27.3)	
Moderate	13 (8.4)	56 (29.9)	132 (26.3)	
Sever	2 (1.3)	53 (28.3)	50 (10.0)	
Gastrointestinal reaction				
Nausea (*n* = 502)				0.000
None	133 (86.4)	116 (62.0)	352 (70.1)	
Mild	15 (9.7)	41 (21.9)	84 (16.7)	
Moderate	5 (3.2)	18 (9.6)	49 (9.8)	
Sever	1 (0.6)	12 (6.4)	17 (3.4)	
Transient visual disturbances				0.049 **
None	144 (93.5)	160 (85.6)	444 (88.4)	
Mild	9 (5.8)	18 (9.6)	45 (9.0)	
Moderate	1 (0.6)	9 (4.8)	9 (1.8)	
Sever	0 (0.0)	0 (0.0)	4 (0.8)	
Vomiting				0.045 **
None	153 (99.4)	171 (91.4)	478 (95.2)	
Mild	1 (0.6)	7 (3.7)	14 (2.8)	
Moderate	0 (0.0)	7 (3.7)	8 (1.6)	
Sever	0 (0.0)	2 (1.1)	2 (0.4)	
Diarrhea				0.067 **
None	145 (94.2)	163 (87.2)	437 (87.1)	
Mild	9 (5.8)	17 (9.1)	40 (8.0)	
Moderate	0 (0.0)	7 (3.7)	19 (3.8)	
Sever	0 (0.0)	0 (0.0)	6 (1.2)	
Burning sensation of eye				0.076 **
None	147 (95.5)	163 (87.2)	444 (88.4)	
Mild	7 (4.5)	14 (7.5)	36 (7.2)	
Moderate	0 (0.0)	8 (4.3)	13 (2.6)	
Sever	0 (0.0)	2 (1.1)	9 (1.8)	
Cardiopulmonary reactions				
Difficulty in breathing				0.017 **
None	145 (94.2)	160 (85.6)	429 (85.5)	
Mild	8 (5.2)	20 (10.7)	58 (11.6)	
Moderate	1 (0.6)	5 (2.7)	15 (3.0)	
Sever	0 (0.0)	2 (1.1)	0 (0.0)	
Palpitation				0.000
None	137 (89.0)	137 (73.3)	384 (76.5)	
Mild	11 (7.1)	26 (13.9)	87 (17.3)	
Moderate	6 (3.9)	15 (8.0)	26 (5.2)	
Sever	0 (0.0)	9 (4.8)	5 (1.0)	
Sore throat				0.046
None	132 (85.7)	139 (74.3)	374 (74.5)	
Mild	16 (10.4)	39 (20.9)	92 (18.3)	
Moderate	4 (2.6)	4 (2.1)	26 (5.2)	
Sever	2 (1.3)	5 (2.7)	10 (2.0)	
Others (minors)				
insomnia	1 (33.3)	0 (0.0)	9 (31.0)	0.387 **
lymphadenopathy	0 (0.0)	1 (33.3)	3 (10.3)	
somnolence	2 (66.7)	1 (33.3)	3 (10.3)	
metallic taste	0 (0.0)	0 (0.0)	4 (13.8)	
joint pain	0 (0.0)	1 (33.3)	6 (20.7)	
backpain	0 (0.0)	0 (0.0)	4 (13.8)	
Use of analgesia (*n* = 835)				0.000
Yes	58 (39.2)	156 (83.4)	338 (67.6)	
No	90 (60.8)	31 (16.6)	162 (32.4)	

** Fisher exact test.

**Table 3:** Analgesics used and physician consultation of the study respondents.

Variables	Vaccine Type			*p* Value
	Sinopharm	AstraZeneca	Pfizer	
Antalgics used (*n* = 533)				0.632
paracetamol	52 (94.5)	136 (87.7)	294 (91.0)	
diclofenac	2 (3.6)	6 (3.9)	7 (2.2)	
iboprofen	0 (0.0)	11 (7.1)	16 (5.0)	
mfenamic acid	1 (1.8)	1 (0.6)	3 (0.9)	
aspirin	0 (0.0)	1 (0.6)	1 (0.3)	
meloxicam	0 (0.0)	0 (0.0)	2 (0.6)	
Physician consultation				0.344
Yes	9 (5.8)	19 (10.2)	45 (9.0)	
No	145 (94.2)	168 (89.8)	457 (91.0)	

## Data Availability

Not applicable.
